# Role of Radiation Therapy in Modulation of the Tumor Stroma and Microenvironment

**DOI:** 10.3389/fimmu.2019.00193

**Published:** 2019-02-15

**Authors:** Hari Menon, Rishab Ramapriyan, Taylor R. Cushman, Vivek Verma, Hans H. Kim, Jonathan E. Schoenhals, Cemre Atalar, Ugur Selek, Stephen G. Chun, Joe Y. Chang, Hampartsoum B. Barsoumian, Quynh-Nhu Nguyen, Mehmet Altan, Maria A. Cortez, Stephen M. Hahn, James W. Welsh

**Affiliations:** ^1^Departments of Radiation Oncology, University of Texas MD Anderson Cancer Center, Houston, TX, United States; ^2^Department of Radiation Oncology, Allegheny General Hospital, Pittsburgh, PA, United States; ^3^Department of Radiation Medicine, School of Medicine, Oregon Health and Sciences University, Portland, OR, United States; ^4^Medical School, University of Texas Southwestern, Dallas, TX, United States; ^5^Department of Radiation Oncology, School of Medicine, Koç University, Istanbul, Turkey; ^6^Thoracic/Head and Neck Medical Oncology, Houston, TX, United States; ^7^Experimental Radiation Oncology, The University of Texas MD Anderson Cancer Center, Houston, TX, United States

**Keywords:** radiation therapy (radiotherapy), immunotherapy, stroma, cancer, tumor microenvironment

## Abstract

In recent decades, there has been substantial growth in our understanding of the immune system and its role in tumor growth and overall survival. A central finding has been the cross-talk between tumor cells and the surrounding environment or stroma. This tumor stroma, comprised of various cells, and extracellular matrix (ECM), has been shown to aid in suppressing host immune responses against tumor cells. Through immunosuppressive cytokine secretion, metabolic alterations, and other mechanisms, the tumor stroma provides a complex network of safeguards for tumor proliferation. With recent advances in more effective, localized treatment, radiation therapy (XRT) has allowed for strategies that can effectively alter and ablate tumor stromal tissue. This includes promoting immunogenic cell death through tumor antigen release to increasing immune cell trafficking, XRT has a unique advantage against the tumoral immune evasion mechanisms that are orchestrated by stromal cells. Current studies are underway to elucidate pathways within the tumor stroma as potential targets for immunotherapy and chemoradiation. This review summarizes the effects of tumor stroma in tumor immune evasion, explains how XRT may help overcome these effects, with potential combinatorial approaches for future treatment modalities.

## Introduction

Cancer therapy has advanced greatly over the past several decades, and recent advances in immunotherapy have led to marked improvement in outcomes and quality of life in patients with cancers previously thought to be incurable (1, 2). However, responses to immunotherapy are not as robust as previously hoped. This has led to increased interest in the mechanisms of tumor immune evasion. Increasing observations strongly suggest the tumor microenvironment (TME) and stroma are sources for tumor evasion of the immune system and related immunotherapies.

The stromal microenvironment of a tumor presents an underlying challenge to the efficacy of cancer immunotherapy. In their seminal review, Hanahan and Weinberg named evading immune destruction as an emerging hallmark of cancer among other related activities, such as metabolic reprogramming and induction of angiogenesis within the TME (3). For cytotoxic T cells and other immune cells to kill cancer cells, physical cell-to-cell contact is necessary (4). However, stromal cells actively orchestrate resistance to antitumor immunity by restricting T cells from making physical contact with cancer cells (5). The stroma surrounding tumor islets of solid malignancies consists of a myriad of molecular and cellular components: immune cells, including myeloid-derived suppressor cells (MDSCs), tumor-associated macrophages (TAMs), and regulatory T cells (Tregs); fibroblasts; epithelial cells; extracellular matrix (ECM) proteins; blood and lymphatic vessels; and various metabolites, chemokines, and cytokines.

Leveraging the components of the stromal microenvironment, tumors employ a variety of strategies for immune evasion. These strategies can be broadly grouped thematically into the following categories: immune cell regulation, metabolic reprogramming, and hypoxia. These immune evasion strategies collectively synergize to blunt the efficacy of tumor-infiltrating lymphocytes (TILs) with regard to both activation and infiltration. Clinically, this may significantly limit significant limitation of cancer immunotherapy. Indeed, evidence suggests that baseline infiltration of both T cells and natural killer cells as well as expression of various chemokines involved in immune cell recruitment to the TME are strongly associated with prognosis for a variety of histological types of cancer (6). Therefore, we believe that the stroma is an underexplored target for immunotherapies that can also synergize with other therapeutic modalities, such as radiation therapy (XRT). Overcoming the immune-suppressive stroma may prove to be integral to unleashing the full potential of immunotherapy and bolstering its antitumor effects.

Radiation therapy is a gold standard of cancer treatment, with more than 50% of cancer patients needing local therapy with XRT (7). With increasing knowledge of the TME's role in immune evasion, interest in the effect of XRT on the TME is growing. From increasing tumor antigen presentation to facilitating trafficking of T cells, XRT plays an important immunogenic role in treatment of cancer and its microenvironment. In this review, we describe how the stroma affects antitumor immunity, XRT's role in disrupting the tumor stroma and TME, and future role of XRT combined with immunotherapy to enhance antitumor immunity.

## Tumor Stroma: Evading the Antitumor Immune Response

### Exclusion of Effector Immune Cells From the Tumor Microenvironment

The most observable effect of the tumor stroma in the context of cancer immunotherapy is the exclusion of T cells from tumor beds, resulting in a “cold” phenotype. Inflammatory chemokines are the primary factors involved in trafficking and homing of T cells to the TME. Gene expression profiling performed with a series of melanoma metastases identified six chemokines—CCL2, CCL3, CCL4, CCL5, CXCL9, and CXCL10—that are associated with CD8^+^ T-cell recruitment and demonstrated that chemokine blockade inhibited migration of CD8^+^ effector T cells *in vivo* (8). To induce rapid chemotaxis toward inflammatory chemokines, activated T cells have increased expression of surface chemokine receptors, including CXCR3, which, along with its interferon (IFN)-γ-inducible ligands, has been associated with a Th1 immune response and accumulation of both T and natural killer cells in the tumor bed (9–11).

However, tumors commonly dysregulate normal chemokine pathways and express different chemokines, such as nitrosylated CCL2 and CCL28, which result in the recruitment and accumulation of Tregs, TAMs, immature dendritic cells (DCs), and MDSCs and form an immune-suppressive TME (12). TME conditions are partly responsible for such changes in chemokine networks. Nitrosylation of CCL2, which normally supports tumor-infiltrating lymphocyte trafficking into the tumor core, occurs through the production of reactive nitrogen species in the TME (13). CCL28 is produced as a result of tumor hypoxia and the release of damage-associated pattern molecules (14). In addition, tumors often specifically target chemokines that are responsible for cytotoxic T lymphocyte (CTL) infiltration. One such chemokine is CXCL11, which specifically attracts CXCR3^+^ CD8^+^ cells and undergoes proteolytic alterations induced by the tumor, resulting in failure to attract TILs (15). In addition, preclinical and clinical evidence has demonstrated that expression of CCL27, which also plays a role in T-cell homing under inflammatory conditions, is downregulated by hyper-activation of the epidermal growth factor receptor (EGFR)/Ras/mitogen-activated protein kinase (MAPK) signaling pathway in melanoma (16). Overall, manipulation of chemokine networks in the TME results in an abundance of M2 TAMs and other regulatory components that blunt the antitumor activity of CTLs.

In the stroma, both tumor cells and these abundant M2 TAMs secrete various molecules, such as vascular endothelial growth factor (VEGF), interleukin (IL)-10, transforming growth factor (TGF)-β, adenosine, and prostaglandin E_2_, that inhibit DC activation and maturation and suppress the activity of CTLs and natural killer-mediated immunity (17). For example, the production of VEGF, which is a well-known mediator of angiogenesis, can play a strong role in preventing DC precursors from maturing into DCs (18). Likewise, prostaglandin E_2_ secretion modulates chemokine production in favor of Tregs and MDSCs differentiation while inhibiting CTLs and natural killer cell populations and decreases production of IL-2 and IL-12 (19). M2 TAMs have immune-suppressive roles that extend beyond the production of soluble factors. The “immune-excluded” phenotype can physically occur via long-lasting interactions between CTLs and TAMs. Peranzoni and colleagues showed that stromal macrophages impede CD8^+^ T cells from reaching tumor islets by making long-lasting contacts that reduce T-cell motility (20). Upon pharmacological depletion of TAMs, T-cell infiltration and migration into the tumor islets were no longer impeded, and this enhanced the efficacy of anti-programmed cell death protein 1 (PD-1) immunotherapy (20). Clinically, the same study found that lung squamous cell carcinoma patients with high tumor: stroma ratios, which reflected increased CD8^+^ T-cell infiltration into tumor islets, had better overall survival than did patients with low ratios (20).

**Figure 1:**
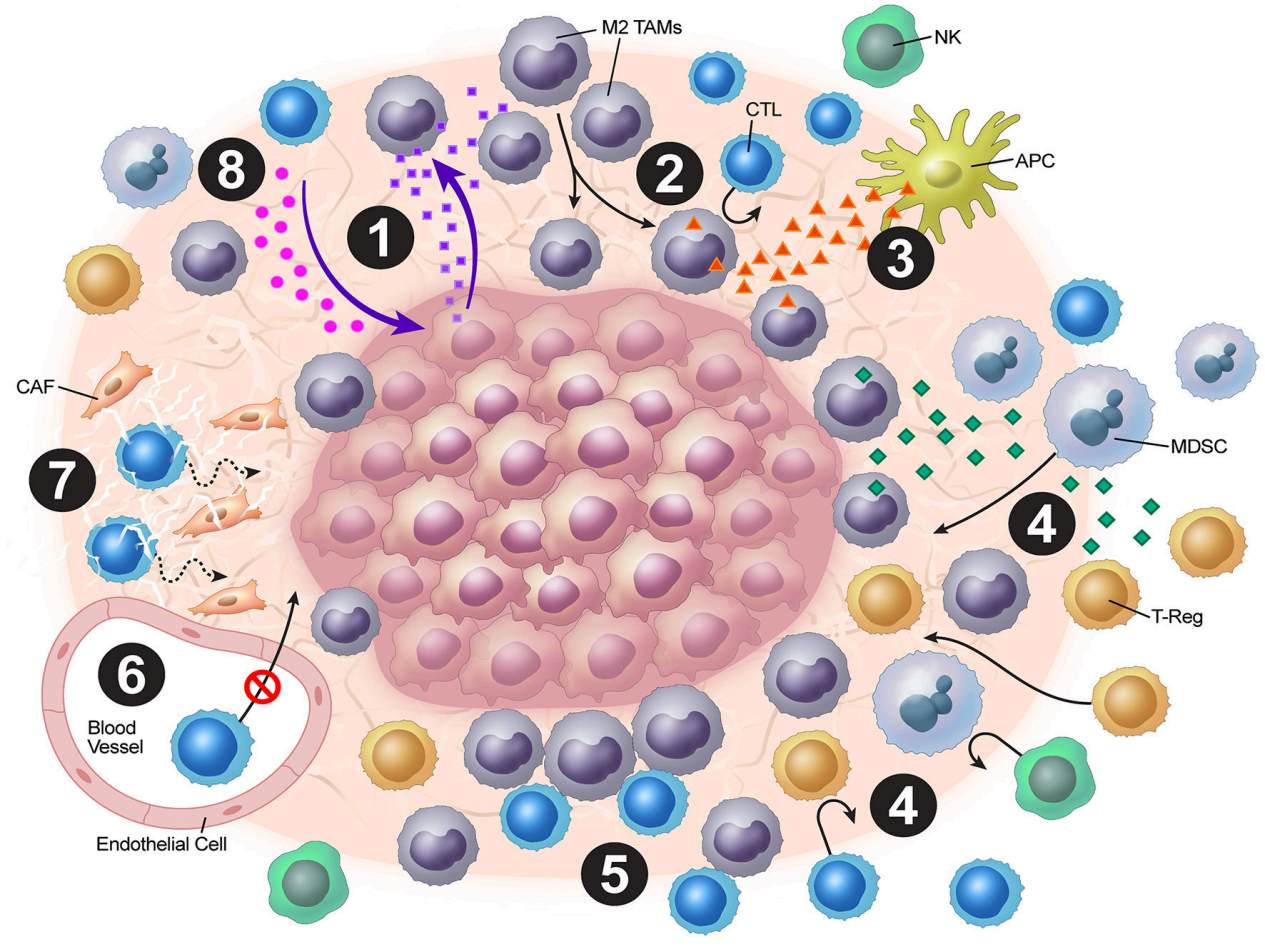
Overview of tumor stromal mechanisms of immune evasion. (1) The tumor stroma disrupts normal chemokine pathways. (2) Chemokine dysregulation leads to increased M2 TAM populations. (3) M2 TAMs release VEGF, which inhibits DC maturation. (4) M2 TAMs also release chemokines and cytokines (e.g. TGF-β), which attract Tregs and MDSCs. (5) Stromal macrophages limit CD8^+^ T-cell infiltration and migration. (6) ICAM and VCAM downregulation lead to decreased CTL penetration. (7) CAFs and the stromal matrix inhibit CTL mobility. (8) Depletion of resources and accumulation of tumor metabolic byproducts leads to blunting of CTL functionality.

Tumor vasculature may play a strong role in the stromal mechanisms of immune exclusion. The migration of T cells through the endothelium, which is often dysregulated as a result of vasculature remodeling, is another challenge to antitumor immunity. For T cells to migrate to the tumor bed, they must adhere to the endothelium (21). However, expression of various endothelial adhesion molecules, such as intercellular adhesion molecule (ICAM)-1 and vascular cell adhesion protein (VCAM)-1, is downregulated in endothelial cells surrounding solid tumors (22). Recently, Motz and colleagues have described a mechanism by which the tumor endothelial barrier regulates T cell migration into tumors (23). In both human and mouse tumor vasculature, the expression of Fas ligand (FasL), which induces apoptosis, was detected, but it was not detected in normal vasculature (23). Additionally, the expression of FasL on endothelium was associated with decreased CD8^+^ infiltration and accumulation of Tregs, which were resistant to FasL due to higher c-FLIP expression. However, this blunting of CD8^+^ T cell infiltration was reversed by pharmacologic inhibition of prostaglandin E_2_ and VEGF, which were shown to cooperatively induce FasL expression on this tumor endothelial “death barrier” (23). The dense stroma matrix architecture also presents a unique challenge to T cell infiltration, and matrix reduction with collagenase has been shown to improve T cell infiltration (24, 25). Finally, cancer-associated fibroblasts (CAFs) in the stroma have pleiotropic roles in secretion of chemokines, cytokines, and metabolites that alter antitumor immunity (26). Molecular strategies to normalize tumor vasculature and induce tertiary lymphoid structures have shown much promise in orchestrating effective T cell immunotherapy preclinically (27–30). Overall, tumor cells employ a combination of these above mechanisms in excluding cytotoxic T-cells from the tumor microenvironment, blunting anti-tumor immunity (Figure 1).

### Metabolic Reprogramming

Metabolic competition between tumor cells and immune cells is known to cause T-cell anergy and immune resistance. Tumor cells, as well as stromal endothelial cells and CAFs, are characterized by the Warburg effect (31). The Warburg effect is traditionally recognized as a unique type of cancer metabolism described as the switch from oxidative phosphorylation to anaerobic glycolysis in the presence of oxygen (32). Warburg found that cancer cells mainly depend on anaerobic glycolysis survival even in the presence of oxygen, which leads to the substantial depletion of glucose from the TME, causing pleiotropic immuno-suppressive effects (33). Excessive depletion of glucose and essential amino acids such as glutamine, tryptophan, and arginine in the TME, coupled with production of metabolites such as lactate, adenosine, and kynurenine, blunts cytotoxic T-cell function while promoting accumulation of regulatory immune cells, such as Tregs, TAMs, and MDSCs (34). Therefore, altered cancer metabolism of tumor stromal cells is a significant factor that mediates resistance of cancer to immunotherapy.

Many metabolic alterations are driven by the need for NADPH, a unique high-energy molecule that is required for lipid synthesis, a building block for the plasma membrane in rapidly growing tumor cells. In almost all types of cancer, both cancer cells and stromal cells like CAFs overexpress transketolase, an enzyme in the pentose phosphate pathway, which importantly produces NADPH and ribose (35). Transketolase is now considered one of the most universally overexpressed genes in cancer metabolism. Additionally, investigators recently found that patients with isocitrate dehydrogenase 1 (IDH1)-mutant glioblastoma had a better prognosis than IDH1 wild type glioblastoma (36). Interestingly, mutation leads to depletion of elevated NADPH pools in cancer cells. Unlike wild-type isocitrate dehydrogenase 1, the mutant form found in glioma patients depletes NADPH pools by converting NADPH to NADP+ (37). Multiple studies have implicated the well-known tumor suppressor p53 in regulating metabolic reprogramming. When Ahmad and colleagues induced overexpression of p53 in human prostate cancer cells and combined it with treatment with 2-deoxy-D-glucose, they showed that the cancer cells overexpressing p53 died of oxidative stress by disrupting glucose influx using 2-deoxy-D-glucose, demonstrating a major role for p53 in glucose metabolism major metabolic switch (38). Their work supports the recently identified role of p53 as a metabolic suppressor of NADPH production (39). One of the enzymes controlled by p53 is malic enzyme, a major NADPH producer in cells (40). Another enzyme whose activity is inhibited by p53 is glucose-6-phosphate dehydrogenase, which is the first step in the pentose phosphate pathway (39). Targeting of cancer metabolism is a crucial consideration in any therapeutic approach and we will later discuss the role XRT plays in this context.

## XRT: Challenging the Tumor Stroma and Tme

### The Evolving Role of XRT in Antitumor Immunity

The field of radiation biology has historically focused on the effects of radiation in killing cancer cells in isolation. Although the earliest cellular radiobiology experiments yielded significant advances in the understanding of DNA damage and repair, they did not account for the impact that local and systemic factors may have on radiation responses. *In vitro* clonogenic and colony formation assays, in which radiation log kill curves were first generated, but did not include an understanding of stromal microenvironment and immunity (41). Moreover, *in vivo* tumor xenograft experiments have historically relied on immune-deficient animal models (42). As such, these classic models that radiation biologists relied on for decades were insufficient to elucidate phenomena such as the abscopal response (43, 44) and the efficacy of PD-1–directed therapies (45).

Increasing evidence has implicated the stromal microenvironment as being a critical mediator of radiation responses both locally and systemically. For example, the observation that COMMA-D cells demonstrate enhanced tumorigenicity when implanted into pre-irradiated fat from murine mammary stroma *in vivo* underlined the hypothesis that radiation can have differential effects on tumors and the surrounding microenvironment (46). Radiation is a potent inducer of vascular injury, inflammation, and fibrosis. Also, hypoxia and activation of hypoxia-inducible factor-1α/VEGF signaling as a result of radiation-induced vascular dysfunction can promote radioresistance (47). Furthermore, irradiation sets in motion a robust inflammatory and fibrotic response in stroma mediated by cytokines such as IL-1, IL-6, IL-10, and TGF-β that can modify tumor responses to both XRT and chemotherapy (48). Indeed, radiation has a myriad of pleiotropic effects in tumors and their stroma that are only starting to be understood (49, 50). With increasing recognition of the fundamental role played by stromal immune signaling in tumor maintenance and radioresistance, pursuing mechanism-based strategies to overcome XRT resistance based on a comprehensive understanding of not only tumor biology but also local stromal and systemic immunobiology is crucial.

Historically, XRT was thought to be primarily immunosuppressive. However, the discovery of the abscopal effect in multiple tumor types (although rare) has significantly altered our understanding of XRT's role in the immune system. This new paradigm demonstrates XRT to be an immunomodulatory tool that facilitates for recruitment and activation of the immune system to fight tumors. The main underpinning of XRT's effect on antitumoral immunity is increasing the release of tumor antigens and their availability for antigen-presenting cells (APCs) to take up and prime T cells. However, XRT also has direct effects on the surrounding stroma that enables the immune system to increase antitumoral responses.

In addition, a potential strategy involves XRT to eradicate all gross disease followed by immunotherapy to eliminate remaining microscopic disease in cancer patients. Researchers demonstrated the benefit of this strategy in the recent PACIFIC trial (NCT02125461) examining sequential XRT and immunotherapy (51). Antonio et al. recently reported results from this randomized phase 3 study of 713 patients with stage 3 locally advanced, unresectable non-small cell lung cancer who received anti-programmed death-ligand 1 antibody, durvalumab, or a placebo after completion of two or more cycles of platinum-based chemoradiation. Recent updated results have demonstrated a markedly longer median progression-free survival (PFS) duration with durvalumab than with the placebo (17.2 vs. 5.6 months) following chemoradiation (52).

We can suggest that XRT and immunotherapy worked synergistically in the PACIFIC trial, in which XRT first ablated all gross disease, leaving behind only microscopic metastases, which immunotherapy controlled. The lack of recurrences in that study, resulting in extended PFS in patients receiving immunotherapy, stems from the enhanced ability of immune cells to infiltrate and eliminate microscopic metastases, which lack a stromal microenvironment but can still seed the growth of larger metastases. Indeed, a study by Zhang and colleagues demonstrated poor prognoses and increased rates of recurrence in non-small cell lung cancer patients with stroma-rich tumors, in whom the tumor: stroma ratio was quantified using hematoxylin-stained tissue specimens (53). Therefore, XRT can ablate all gross disease and its stroma to enhance the effects of immunotherapy on remaining microscopic disease with a less dense stromal microenvironment.

### Immunogenic Mechanisms of XRT

The landmark PACIFIC trial suggests significant improvements in patient outcome by utilizing XRT combined with immunotherapy. This will impact many future trial designs for multiple solid tumors with the goal of improving the patient outcomes. As described previously, XRT was initially used for its direct induction of DNA damage, leading to tumor cell death (54). Historically, this DNA death mechanism was seen as immunosuppressive due to the radiosensitivity of lymphocytes (55). However, with recent advances in technology including stereotactic body radiation therapy (SBRT), which allows for tighter dose distributions and higher doses given, there has been increasing evidence that XRT can serve to help activate T cells and destroy much of the immune inhibitory stroma.

A direct immune-related result of XRT is the release of tumor antigens, which allows for APC presentation and subsequent CD8^+^ cell activation. This modality of cell death is termed immunogenic cell death (ICD). Traditionally, apoptosis is considered a tolerogenic process, which limits the ability of the immune system to develop a full response. However, with ICD, an external stress source facilitates the release of danger-associated molecular patterns (DAMPs), which elicit a signal to APCs and instigate cell death (56). Several DAMPs have been implicated in the ICD pathway, such as CRT, HMGB1, and secreted ATP (57, 58).

Even with increased antigen release due to XRT leading to increased ICD, the TME does not allow for proper activation of the immune response. For example, tumors have demonstrated downregulated major histocompatibility complex class I (MHC-I) expression, which leads to decreased recognition of tumor cells by effector T cells (59, 60). Clinically, increased MHC-I expression has been associated with improved survival of multiple cancer types (61, 62). Biologically, this makes sense, as an increase in the number of T cell-mediated reactions can occur, conferring stronger immune responses. The reduced expression of MHC-1, found biologically is found in the tumor stroma can be overcome by XRT. *In vitro* studies have demonstrated that XRT can upregulate MHC-I expression at sublethal doses (63–66). One underlying mechanism promoting this phenotype occurs through increased peptide availability following XRT and subsequent mammalian target of rapamycin (mTOR) activation, leading to an increase in MHC-I protein subunits in a dose-dependent fashion (67). Ultimately, this leads to an increase in effector activity by facilitating proper effector signaling, thereby increasing the overall T-cell repertoire.

Having antigens and the necessary cell-surface receptors alone is not sufficient to overcome all of the negative effects of tumor stroma on the immune system. Activation of pro-inflammatory signals to overcome the immunosuppressive population of Tregs, M2 TAMs, and MDSCs is imperative. XRT has been shown to facilitate this process through several chemokine/cytokine modulations within the TME. Type I IFNs play a role in this process, as they are required for proper DC maturation, increasing MHC-I expression and T-cell priming (68). IFN expression is upregulated by XRT through the cGAS-STING pathway. In this process, cGAS is activated by the DNA damage caused by XRT, with downstream effects leading to production of nuclear factor-κB and other transcription factors for IFN (69). Indeed, in a recent *in vivo* study using an anti-PD-1 therapy-resistant mouse lung cancer cell line, suppression of type I IFN expression was associated with anti-PD-1 therapy resistance due to reduced MHC-I expression, but tumors became responsive to anti PD-1 therapy after XRT (66).

XRT has also been shown to orchestrate T cell immunotherapy by promoting T-cell homing into the tumor bed through a variety of mechanisms including chemokine expression, macrophage polarization, and expression of adhesion molecules on tumor vasculature. As described previously in this review, the stroma provides signals that prevent trafficking and homing to a tumor using several chemokines. With XRT, these chemokine signals are altered and allow for better lymphocyte “pulling” into the TME. Expression of CXCL16, a chemokine that assists in T-cell infiltration, has been upregulated in breast cancer cells after XRT at 2 fractions at 12 Gy. This allows for increased CD8^+^ activation of T cells expressing CXCR6 *in vivo*. Subsequently, loss of CXCR6 results in loss of this phenotype and poor outcomes *in vivo* (70). Also, immune cell infiltration has occurred with low-dose XRT (2 Gy). Klug et al. demonstrated polarization of M2 TAMs to NOS^+^ M1 TAMs after low-dose XRT (71). Moreover, low-dose XRT can increase T-cell recruitment to pancreatic tumors *in vivo*.

Adhesion molecules are also altered after XRT. Studies of K562 cells have demonstrated upregulation of VCAM-1 expression *in vitro* after exposure to 16–20 Gy within 24 h (72). This upregulation of VCAM-1 has been further observed in other cancer types *in vivo* after low-dose XRT (73). Upregulation of adhesion molecule expression is not limited to tumor cells, as lymphatic endothelial cells have also demonstrated this change after single doses of XRT (74). Furthermore, ICAM-1 expression is increased in several tumor cell lines after XRT (75, 76). Overall, upregulation of these adhesion molecules allows for increased infiltration of lymphocytes to tumor cells, increased affinity binding to CD3^+^ cells, and ultimately increased immunogenicity.

**Figure 2:**
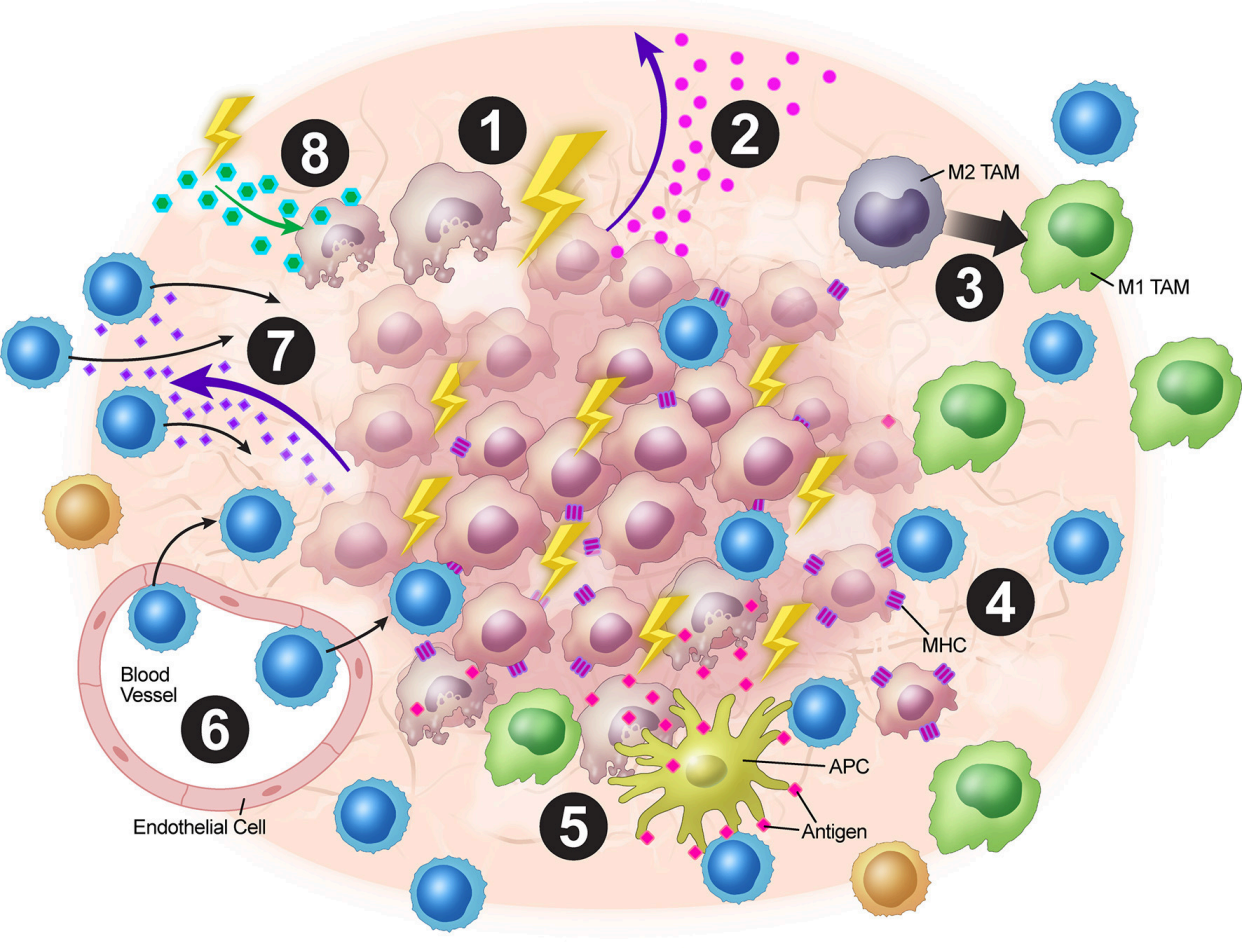
Overview of XRT's effects on the tumor stroma. (1) XRT ablates and reprograms the stroma. (2) Increased STING pathway activation leads to upregulation of type I IFNs. (3) TAMs are polarized from the M2 to the M1 phenotype. (4) Radiation increases MHC-I expression on tumor cells. (5) Tumor destruction leads to increased antigen presentation via ICD. (6) Upregulation of VCAM-1 and ICAM-1 expression leads to increased T-cell adhesion within the stroma. (7) Upregulation of expression of chemokines such as CXCL16 leads to T-cell trafficking into the TME. (8) Radiation alters stromal cell metabolism leading to increased reactive oxygen species and subsequent surrounding cell death due to changes in oxygen requirement.

In a nutshell, XRT leads to neo-antigens and DAMPs release, upregulation of MHC-I, expansion of T-cell repertoire, activation of the STING pathway and production of Type-I interferons, and upregulation of adhesion molecules such as VCAM/ICAM. Additionally, low dose XRT could polarize the M2 macrophages to M1 and reduce the levels of tumor-induced Tregs. Further work is needed to make conclusions regarding the optimal combinations and timings of XRT with immunotherapy and other targeted treatments to overcome immune resistance that is orchestrated by tumor stroma. The tumor stroma is complex and intricately dynamic with multiples layers of cytokine signaling and XRT provides a much-needed tool to combat such a clinical challenge. We believe the above-mentioned mechanisms of XRT work in concert to elicit systemic anti-tumor responses. In summary, XRT has multiple effects on the tumor stroma to increase anti-tumor immunity (Figure 2).

### Targeting Cancer Metabolism

Based on recent discoveries in the field of cancer metabolism that we discussed previously, researchers have proposed new rationales behind cancer metabolism, providing insight into why XRT and immune therapy are perhaps the best clinically available weapons we have to fight cancer. Very little effort has been directed toward tackling the metabolic aspect of cancer using radiation and addressing targeting of immune metabolism to improve cancer therapies (77). XRT is the only effective established clinical tool that takes advantage of the metabolic aspect of cancer. In their 2005 article, Spitz et al. described that when glucose was deprived from cell media culture, cancer cells died of oxidative stress (78). What they showed was that by shutting off the glucose influx into cancer cells, they were unable to manipulate the metabolic environment to fight oxidative stress, which can be induced by XRT. Later, Coller et al. discussed the importance of protection against reactive oxygen species manifested in patients with abnormal cancer metabolism (79).

As of now, the only available clinical tool to induce oxidative stress is XRT, which works by increasing the amount of reactive oxygen species, such as hydroxyl radical, which causes DNA damage and depletes NADPH pools needed for the proliferation of cancer cells. XRT causes oxidative stress to kill cancer cells by effectively depleting the pool of NADPH, which is rapidly consumed by proliferating cancer cells to support their growth, reduce their levels of oxidized glutathione, or neutralize any oxidative damage they go through. Of note, both endothelial cells and fibroblasts demonstrate upregulation of the glycolytic pathway and pentose phosphate pathways, so these stromal cells would be affected by XRT as well.

## XRT Limitations: Stratigies to Overcome Resistance

Radiation provides strong antitumor immunogenic responses to help overcome the anti-tumor immune evading mechanisms that the TME provides. However, the TME also has mechanisms that help tumors evade the full effects of initial and subsequent rounds of XRT. One important mechanism of this evasion comes from fibrosis after XRT. Fibrosis, which is initiated by the activation of inflammatory pathways, allows for further radioprotection and decreased vascular permeability of tumors which lead to increased resistance to subsequent therapies (50). CAFs are one of the largest cell populations within the tumor stroma that are drivers of stromal proliferation (80). After XRT, these CAFs are further activated. This additional activation enables CAFs to produce several cytokines, proteins, and enzymes to promote stromal expansion (80, 81). *In vivo* studies demonstrated that CAFs can mediate autophagy and irradiated tumor cell recovery through insulin-like growth factor 1-mediated mechanisms (82).

CAFs also produce important proteins such as collagen, fibronectin and integrins. Studies have demonstrated integrins to be of particular interest following XRT. A modest post-XRT increase in expressions of both α and β integrins within the stroma occurred *in vivo* (83, 84). These integrins help anchor tumors in place as well as initiate integrin-specific signal transduction. These effects lead to and promote chemoradiation resistance of tumors and induce tumor growth for multiple cancer types (85). Mantoni et al. demonstrated this association in pancreatic cancer cases, as cancer cells co-cultured with irradiated fibroblasts demonstrated greater radioresistance and integrin concentration than did their non-irradiated counterparts (86). Integrins are also implicated to have roles in tumor invasion and metastasis (87). Clinically, integrin expression is strongly associated with radioprotection and increased proliferation of breast cancer (88). Mechanistically, how integrins enforce this phenotype has yet to be determined. One *in vivo* study demonstrated that β_1_-integrins produced inhibitory signals in an insulin-like growth factor 1 receptor-dependent manner in irradiated prostate tumors (84). Another study demonstrated that radioresistance develops in small-cell lung cancer cells through β_1_-integrin–mediated phosphatidyl inositol 3-kinase activation (89).

Inhibition of these CAFs is an area of active investigation. Tirosh et al. sought to elucidate genotypic and phenotypic states of melanomas using single-cell RNA sequencing of tumor samples from patients with metastatic disease (90). They found that the enzyme NADPH oxidate 4 (NOX4), is an integral component of fibroblast differentiation and may be a viable target for inhibition of CAF-associated tumor immune evasion. Although multiple phase 1/2 trials have demonstrated CAF inhibitors to be safe, they did not demonstrate improved tumor control or survival in patients with metastatic colorectal or pancreatic cancer (91–93). Notably, none of these trials included patients receiving XRT. In theory, combination of CAF inhibitors with XRT will minimize immunosuppression and maximize anti-tumorigenicity.

Another important aspect of the TME is the presence of Tregs, which suppress immunity through a variety of mechanisms, including TGF-β and IL-10 production, IL-2 depletion, and ATP degradation into immunosuppressive adenosine via the ectoenzymes CD39 and CD73 (94). Importantly, Tregs are known to correlate with poor prognosis for various cancer subtypes (95). When a tumor is irradiated, various changes in its Treg population occur. Researchers showed that Tregs appear to be more radioresistant than other subsets of T cells, thus increasing the prevalence of Tregs at a tumor site (96, 97). Muroyama et al. further demonstrated Treg proliferation with increased Ki-67 staining for Tregs after XRT when compared to control (98). In addition, the authors blocked T-cell migration into tumors using fingolimod and saw similar results, suggesting that the Tregs at tumors proliferate. In a different study, 8.5 Gy given five times decreased the population of Tregs and their suppressive capabilities (99). These studies suggest that different doses of radiation can have different effects on the Treg population, with hypofractionation perhaps having more anti-Treg effects than single doses.

Thus, depletion of Tregs in combination with XRT is a logical antitumor strategy. Schoenhals et al. investigated the effects of an IgG2a (depleting isotype) anti-glucocorticoid-induced tumor necrosis factor-related protein (GITR) antibody in an anti-PD-1–resistant murine lung adenocarcinoma model (100). They found that the protein was highly expressed at the tumor site, that anti-GITR therapy preferentially depleted Tregs at the tumor site, and that combining this therapy with XRT and anti-PD-1 therapy generated a systemic and durable antitumor response. These results highlight the potential of XRT to overcome treatment resistance of cancer, an area of intense interest in the field of cancer immunotherapy.

MDSCs are also are also believed to be among the main drivers of TME immunosuppression, and their presence has been correlated with poor prognosis and response rates for many types of human tumors (101). Studies demonstrated that the frequency of circulating MDSCs was higher with increased tumor burden for multiple solid tumors (101–105). Also, XRT has been shown to inhibit MDSC infiltration into the TME via CCR2 blockade (106). Studies have shown the administration of low-dose gemcitabine depletes MDSCs at low doses in murine models (107). Clinical studies of the safety and efficacy of combined low-dose gemcitabine and anti-PD-1 therapy (NCT03302247) are currently underway. Given the synergy between anti-PD-1 therapy and XRT described above, the addition of XRT to this dual therapy may further improve outcomes.

In addition to these immunosuppressive cells, XRT can impact the fitness of CD8+ effector T cells through cytokines. Interferons have been found to play a role in signaling for T cell exhaustion through programmed-death ligand 1 (PD-L1), which is a member of the B7 superfamily (68, 108). One study found IFNγ is produced after hypofractionated XRT doses with a subsequent increase in PD-L1 expression *in vivo* (109). They found that when combined with anti-PD-L1 immunotherapies, T cells can be rescued from this exhaustive phenotype. Clinically, anti-PD-L1 therapies have recently shown promise, with the ES-SCLC patients demonstrating significant survival benefit with atezolizumab in addition to standard-of-care chemotherapy (110). Given this information together, clinical trials with anti-PD-L1 immunotherapy and XRT may bear even more pronounced results.

Hypoxia also poses a challenge for XRT and its ability to ablate tumors and recruit effector immune cells within the TME. MDSCs and TAMs are heavily recruited to these hypoxic environments through various mechanisms, including colony-stimulating factor 1, VEGF, endothelin, and several other proteins (111). Within the tumor stroma, TAMs have plasticity in their phenotype. As noted above, M1 TAMs are characterized as pro-inflammatory and encourage anti-tumoral responses, whereas M2 TAMs are anti-inflammatory and encourage tumor growth. The distribution of these macrophages within the tumor stroma mirrors their stimuli. Specifically, hypoxic conditions promote the tumoral production of IL-4, IL-10, and TGF-β, which promote M2 polarity and attenuate proper anti-tumoral immune responses (112). *In vivo* studies of prostate cancer demonstrated that M2 TAMs which express arginase-1 and COX-2 are recruited to these hypoxic centers after irradiation and promote tumor growth (113, 114). At higher populations and stronger signaling compared to M1 TAMs and other immunoproliferative cells, these immunosuppressive cells dampen the effector anti-tumor immunity within the stroma after XRT.

Overall, the evidence that XRT modulates the TME and the balance between pro-tumoral and antitumoral signaling is substantial. Investigators have placed an emphasis on how fractionation and dosing play a role in these changes (115, 116). However, from a broader perspective, even with XRT's greater immunogenic capabilities through increased antigen release and ICD, the overall number of cases in which true abscopal effects are seen has been limited (44). Further studies are warranted to evaluate the impact of dosing on these immunogenic characteristics of XRT.

Additionally, the practicality of XRT in the setting of systemic disease is uncertain given increased time demands. For example, the time on the therapy table for each patient per isocenter would increase dramatically. Also, the precision required to target multiple isocenters is not yet possible even with the currently available SBRT technology. Given these practical and mechanistic limitations, our current understanding of XRT as monotherapy for systemic disease is limited. Further studies are warranted to evaluate the timing, dosing, and tolerance of multisite irradiation.

Meanwhile, given the current landscape of multi-agent immunotherapy, such as the combination of anti-PD-1 and anti-CTLA-4 therapy, the body of literature on the synergy of XRT and immunotherapies is rapidly growing (117–119). Targeting immunosuppressive cell populations upregulated by XRT, such as CAFs, Tregs, MDSCs, and TAMs, may further enhance systemic responses to combined XRT-systemic treatment strategies. Future studies must build upon our translational knowledge of these critical relationships to incorporate into clinical trials.

## Future Considerations and Goals

The rationale for combining XRT and immunotherapy is clearly apparent based on the aforementioned synergistic mechanisms. This is exemplified by the rapidly increasing number of ongoing prospective trials of combined-modality therapy for cancer (120, 121). Although a few of these are phase 3 studies, most are phase 1/2 trials given little, low-quality evidence of the safety and efficacy of combined immunotherapy and XRT for cancer at various sites (122–125).

The construction of these prospective investigations has several implications for the design of future studies. Although many of the trials in a previous systematic review evaluated concurrent therapy, few specifically evaluated the risks and benefits of this approach with sequential therapy (120). Mechanistically, as described above, delivering XRT prior to immunotherapy has several theoretical benefits, namely regarding antigen presentation, lack of T-cell depletion from concurrent therapy, and modulation of the TME. However, although some data points to a benefit of XRT delivered prior to immunotherapy (126), other data demonstrates better outcomes if both are given concurrently (127) or immunotherapy is followed by XRT (128). Thus, because most of the aforementioned ongoing trials are phase 1 studies, future phase 2/3 work will be dependent on the paradigm put forth by phase 1 data, researchers sincerely hope that future randomized studies directly evaluate the timing of XRT and immunotherapy (e.g., NCT02525757). However, the effect of their timing is likely dependent on the clinical setting, neoplasm, and/or immunotherapeutic agent.

Future studies must also evaluate combinatorial therapy consisting of XRT and multi-agent immunotherapy as well as chemoimmunotherapy. Although a clear concern is that multi-agent immunotherapy may be more toxic than a single agent alone, multi-agent treatment yields better outcomes than do some single agents as noted in the CheckMate 067 metastatic melanoma trial (129). However, whether additional XRT creates unacceptable toxicity with the use of multiple immunotherapeutic compounds is unknown. Likewise, use of chemoimmunotherapy may increase in the future based on the findings of the KEYNOTE-189 trial, which compared chemoimmunotherapy with chemotherapy alone (130). For most disease sites, although delivering concurrent chemoradiation increases the toxicity over that of a single modality alone, the effect of XRT with chemoimmunotherapy remains unknown and must be addressed.

Just as candidate radiosensitizers have been and continue to be developed for XRT, another goal is to explore candidate immunosensitizers that are not immunotherapeutic compounds but rather promote and stimulate the immune system in ways that allow for enhanced immunotherapy effects while minimizing complication risks to normal tissues, thereby improving the therapeutic ratio. This is important because excessive immune system “drive” may result in potentially lethal toxic effects. Nevertheless, because the response rate for most immunotherapeutic compounds in seminal clinical trials is about 20%, novel biomolecules are needed to increase this rate (131, 132).

Furthermore, the synergy between XRT and immunotherapy may be exemplified by using XRT as a “pseudo-systemic agent” in patients with oligometastatic disease or even widely metastatic disease with good initial responses to chemotherapy and/or immunotherapy (133). Because these patients are expected to survive longer than those with widely disseminated disease, aggressive therapy in them is becoming more reasonable.

Recently, we have seen an increasing trend in the number of positive trials in which XRT is used to treat up to three metastatic sites. Patients with a greater number of metastases would also benefit, but such an approach would be logistically arduous due to the need for multiple isocenters. The development of technologies that make multi-site XRT easier, together with technologies that automate target delineation and treatment planning, such as deep and machine learning, may make XRT more pseudo-systemic in the future, especially when integrating it with other synergistic treatments, such as immunotherapy (134).

## Conclusions

The stroma is an important component of the TME to study because it has significant implications for limiting antitumor immunity. XRT has long been considered to damage cancer cell DNA, but its effects on the stroma have received little consideration. Given reported evidence, one of the greatest benefits of XRT is its ability to eradicate and reprogram the stroma, the same stroma that too often limits the delivery of systemic treatments such as chemotherapy, targeted therapy, immunotherapy, and even cellular therapy. Radiation's ability to eradicate areas of gross disease provides a strong rationale for its use with systemic agents, which would eradicate remaining circulating microscopic disease. Systemic agents are much more effective against the microscopic disease for many reasons, as they no longer face the hypoxic and metabolic changes associated with gross tumor deposits, providing much greater access to target tissues and improving T-cell functionality.

Going forward, we must rationally combine radiation with other stroma-modifying agents, such as colony-stimulating factor 1 receptor, indoleamine 2, 3-dioxygenase 1, and TGF-β inhibitors, to further exploit these advantages. XRT already provides substantial benefits to patients with localized or oligometastatic disease. Combining XRT with immunotherapy will potentially benefits to patients with more advanced metastatic disease and continue to improve survival.

## Author Contributions

All authors contributed to the writing of the manuscript. HM and RR compiled the first draft of the manuscript in preparation for submission. All authors contributed to revisions and subsequent edits.

### Conflict of Interest Statement

JW and JC have received research grants from Bristol-Myers Squibb. JW has also received grants from Varian Medical Systems and OncoResponse, and he is a co-founder of Healios, MolecularMatch, and OncoResponse. In addition, JW is on the scientific advisory boards for RefleXion Medical and Checkmate Pharmaceuticals and receives laboratory research support from Varian Medical Systems, Incyte, Calithera, and Checkmate Pharmaceuticals. JC is a shareholder of Global Oncology One. The remaining authors declare that the research was conducted in the absence of any commercial or financial relationships that could be construed as a potential conflict of interest.
